# Evaluation of Kidney Donor Risk Index/Kidney Donor Profile Index as Predictor Tools of Deceased-Donor Kidney Transplant Outcomes in a Greek Cohort

**DOI:** 10.3390/jcm12062439

**Published:** 2023-03-22

**Authors:** Maria Darema, Diamanto Athanasopoulou, Ioannis Bellos, Ioanna Tsoumbou, Angeliki G. Vittoraki, John Bokos, Smaragdi Marinaki, Ioannis N. Boletis

**Affiliations:** 1 Department of Nephrology and Kidney Transplantation, Medical School, Laiko General Hospital of Athens, National and Kapodistrian University, 11527 Athens, Greece; 2 Immunology Department & National Tissue Typing Center, General Hospital of Athens “G. Gennimatas”, 11527 Athens, Greece; 3 Transplantation Unit, Laiko General Hospital, 11527 Athens, Greece

**Keywords:** KDPI, KDRI, kidney transplantation, deceased-donor, graft survival

## Abstract

The Kidney Donor Risk Index (KDRI) and Kidney Donor Profile Index (KDPI) have been developed to assess deceased-donor graft quality, although validation of their utility outside the USA remains limited. This single-center retrospective cohort study evaluated the ability of KDRI and KDPI to predict transplant outcomes in a Greek cohort. The efficacy of KDRI, KDPI, and donor’s age in predicting death-censored graft failure was primarily assessed. Overall, 394 donors and 456 recipients were included. Death-censored graft survival was significantly worse with increasing KDRI (hazard ratio—HR: 2.21, 95% confidence intervals—CI: 1.16–4.22), KDPI (HR: 1.01, 95% CI: 1.00–1.02), and donor’s age (HR: 1.03, 95% CI: 1.00–1.05). The unadjusted discriminative ability was similar for KDPI (C-statistic: 0.54) and donor’s age (C-statistic: 0.52). The KDPI threshold of 85 was not predictive of graft failure (*p*-value: 0.19). Higher KDPI was linked to delayed graft function and worse kidney function, but not among expanded-criteria donor transplantations. No significant association was found between KDRI, KDPI, and patient survival. In conclusion, increasing KDRI and KDPI are linked to worse graft function, although their ability to discriminate long-term graft failure remains limited.

## 1. Introduction

End-stage kidney disease (ESKD) represents a rising global health concern, associated with impaired quality of life and high mortality rates [1]. Kidney transplantation provides a substantial survival benefit over dialysis [2], although no country is currently able to meet its kidney allograft demands [3]. The evaluation of kidney graft quality constitutes a critical step to effectively increasing the donor pool, optimizing the allograft allocation process, and improving transplant outcomes. A binary classification of deceased donors had been proposed by the UNOS (United Network for Organ Sharing) in 2002, categorizing them as standard and expanded criteria, with the latter group conferring a 1.7-fold higher risk of graft failure [4]. However, this dichotomy has been criticized due to its inadequacy in reflecting the true variability of graft quality [5].

In this context, the OPTN (US Organ Procurement and Transplantation Network) adopted a new kidney allocation system in 2014, aiming to ameliorate the donor–recipient longevity matching. To this end, the KDPI (Kidney Donor Profile Index) has been developed as a mapping of the Kidney Donor Risk Index (KDRI), based on 10 donor parameters, expressing the relative graft failure risk for a given donor compared to the risk attributed to the average donor from the prior calendar year [6]. To enable graft allocation, the KDPI is applied in conjunction with the EPTS (Estimated Post Transplant Survival) score, which is assigned to the waitlisted candidates and is predictive of recipient survival after kidney transplantation [7].

To date, the KDRI and KDPI lack wide external validation in populations outside the USA and their implementation in non-US patients may be complicated by significant differences in transplant programs, donor age, and comorbidities across regions [8]. The present study aims to examine the utility of donor KDRI and KDPI in the prediction of post-transplant outcomes in the Greek population. To achieve this, their discriminative ability is tested by investigating their association with long-term graft and recipient survival, both in standard and expanded criteria donor transplantations.

## 2. Materials and Methods

### 2.1. Study Design and Population

This was a single-center retrospective cohort study, conducting a chart review of all deceased-donor kidney-only transplantations that were performed in Laiko General Hospital, Athens, Greece, between 1 January 2008 and 31 December 2018. Living-donor transplantations and cases with missing data in study outcomes were excluded. Kidney transplant recipients were actively followed up until December 31, 2021. For losses to follow-up, relative data were retrieved through medical records and hospital discharges.

Donors were categorized as standard-criteria donors (SCD) and expanded-criteria donors (ECD). Specifically, ECD were defined as those with an age greater than 60 years or as those aged between 50 and 60 years, fulfilling at least two of the following criteria: history of arterial hypertension, serum creatinine above 1.5 mg/dL, and cerebrovascular accident as the cause of death [9].

### 2.2. Data Collection

The following donor characteristics were collected: age, sex, ethnicity, body mass index (BMI), history of arterial hypertension and diabetes mellitus, hepatitis C status, serum creatinine, cause of death, and donation after circulatory death. BMI was calculated as weight (in kilograms) divided by the square of height (in meters). The main exposures were the values of the donor KDRI (Kidney Donor Risk Index) and KDPI that were compared to the donor age alone in regard to graft function and survival after transplantation. Specifically, the KDRI score was estimated based on ten donor characteristics as suggested by the OPTN. The donor characteristics used in the estimation of KDRI are presented in Table A1. KDRI is calculated by summing the components and applying the antilog function. KDPI is expressed as a cumulative percentage that represents a mapping of KDRI, ranging from 0 to 100% [7]. KDPI was calculated according to the 2020 conversion table, which is based on the reference population of all deceased donors recovered for the purpose of transplantation in the U.S. in 2020 [10].

The main collected baseline recipients’ characteristics were the following: age, sex, BMI, diabetes mellitus status, duration of dialysis, and EPTS score. The EPTS score ranges from 0 to 100%, with lower values predicting longer graft survival [11]. The induction and maintenance immunosuppression regimens were also recorded. The primary outcome of the study was death-censored graft survival. Secondary endpoints included delayed graft function (DGF), estimated glomerular filtration rate (eGFR) in the 1st, 3rd, and 5th post-transplant year, and overall patient survival. DGF was defined as the use of dialysis during the first post-transplant week. The eGFR was calculated using the 2009 CKD-EPI (Chronic Kidney Disease Epidemiology Collaboration) formula [12]. For the death-censored graft survival estimation, the follow-up period was censored at the time of death in the case of death with a functioning graft.

### 2.3. Statistical Analysis

Statistical analysis was conducted in R-4.0.5 (main packages “survival” [13] and “survminer” [14]). Statistical significance was defined using the *p*-value threshold of 0.05. The normality of continuous variables was examined by the visual inspection of histograms, along with the significance of the Kolmogorov–Smirnov test [15]. For the description of variables considered as normally distributed, the mean and standard deviation were used and comparisons were performed with the Student’s *t*-test; otherwise, the median and interquartile range (IQR) were reported and the non-parametric Mann–Whitney U-test was applied to enable comparisons [16]. For the analysis of categorical variables, the chi-squared test was used; when its assumptions were not met, the Fischer’s exact test was implemented [17]. For the endpoint of eGFR, linear regression analysis was applied, while the outcomes of death-censored graft survival and patient survival were assessed by Cox proportional hazards regression analysis. Both univariable and multivariable models were fitted, adjusting for recipients’ age, sex, ΒΜΙ, dialysis vintage, EPTS score, induction, and maintenance immunosuppression. The predictive ability of models was evaluated with Harrell’s C-statistic [18], which is a rank correlation measure adapted for censored survival data. KDPI and KDRI were analyzed both as continuous variables and as categorical ones by dividing them into quartiles. The KDPI cut-off of 85 was also defined a priori based on previous literature [6] and was used in the analysis. Subgroup analysis was performed by separately examining transplantations from SCD and ECD. Kaplan–Meier curves were constructed and compared with the log-rank test.

### 2.4. Ethics Statements

The research protocol was approved by the local ethical committee (Laiko General Hospital, National and Kapodistrian University of Athens, Greece) and was in accordance with the principles of the Declaration of Helsinki. All recipients were fully informed about the study procedures and provided written informed consent.

## 3. Results

### 3.1. Study Population

**Figure 1:**
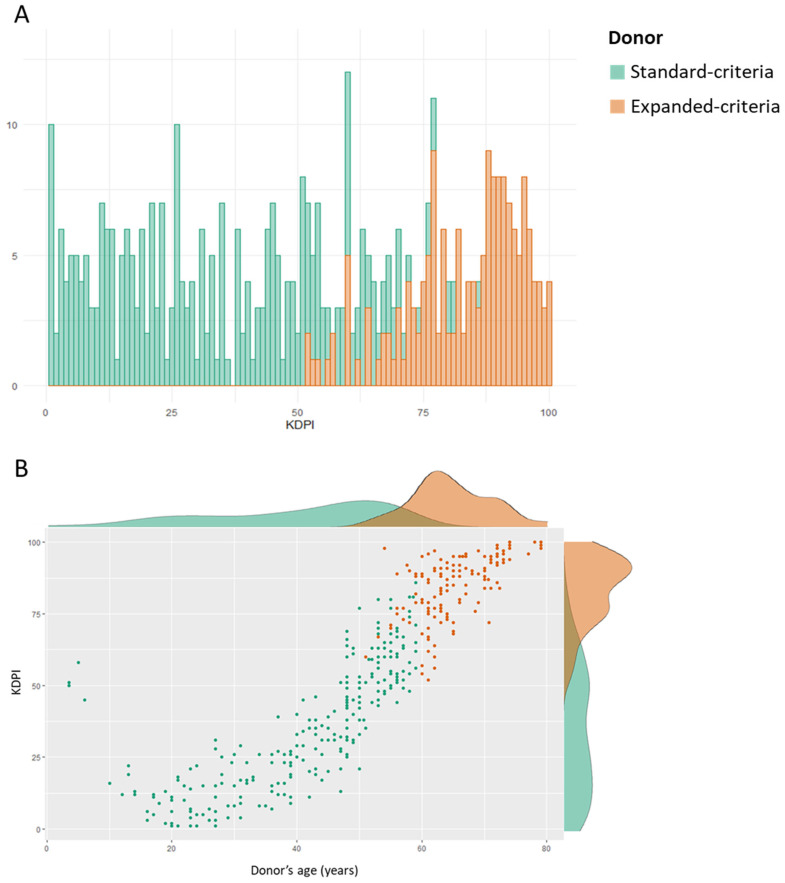
Distribution of KDPI among standard-criteria and expanded-criteria donors (**A**). Scatterplot showing the relationship between donor age and KDPI. Donor age was significantly correlated to higher KDPI (Spearman *ρ*: 0.924, *p*-value < 0.001) (**B**).

The analysis was based on a total of 394 (251 SCD and 143 ECD) kidney donors and 456 recipients. The main patients’ baseline characteristics are summarized in Table 1. The median donor age was 53 years (IQR: 39.3 to 61), while 111 donors were older than 60 years. A KDPI over 85 was noted in 77 donors (76 ECD, 1 SCD). ECD presented significantly older age, greater BMI, as well as higher percentages of males and patients with diabetes mellitus or hypertension. The donors had a median KDRI of 1.02 (IQR: 0.78 to 1.34, range: 0.37 to 2.41) and KDPI of 54 (IQR: 25 to 79, range: 1 to 100), with both values being significantly higher among ECD ones. The distribution of KDPI in SCD and ECD, as well as the correlation between donor age and KDPI is schematically illustrated in Figure 1. In SCD, KDPI ranged from 1 to 86, while in ECD, from 52 to 100; hence, 61 SCD presented KDPI scores greater than 52, overlapping with KDPI values of ECD. Donor age was strongly correlated to significantly higher KDPI (Spearman *ρ*: 0.924, *p*-value < 0.001).

The median age of kidney recipients was 52 years (IQR: 43 to 59) and was higher among ECD recipients. The main induction therapy was basiliximab (74.6%), while the most commonly administered maintenance immunosuppression regimen was the combination of mycophenolate mofetil, a calcineurin inhibitor, and corticosteroids (87.3%). No significant difference in EPTS score was noted between SCD and ECD. The median follow-up period was 6.3 years (IQR: 3.6 to 10).

### 3.2. Delayed Graft Function

Overall, DGF complicated the course of 169 cases. No significant difference in DGF rates was observed between SCD and ECD transplantations (31.7% vs. 46.2%, *p*-value: 0.558). DGF was linked to significantly higher KDPI (58 vs. 51, *p*-value: 0.045) and KDRI (1.04 vs. 1.00, *p*-value: 0.045) scores. Moreover, in standard-criteria donor transplantations, DGF was associated with significantly greater values of both KDPI (40 vs. 26, *p*-value: 0.011) and KDRI (0.89 vs. 0.79, *p*-value: 0.009), while no significant associations were observed in ECD transplantations.

### 3.3. Kidney Function

**Figure 2:**
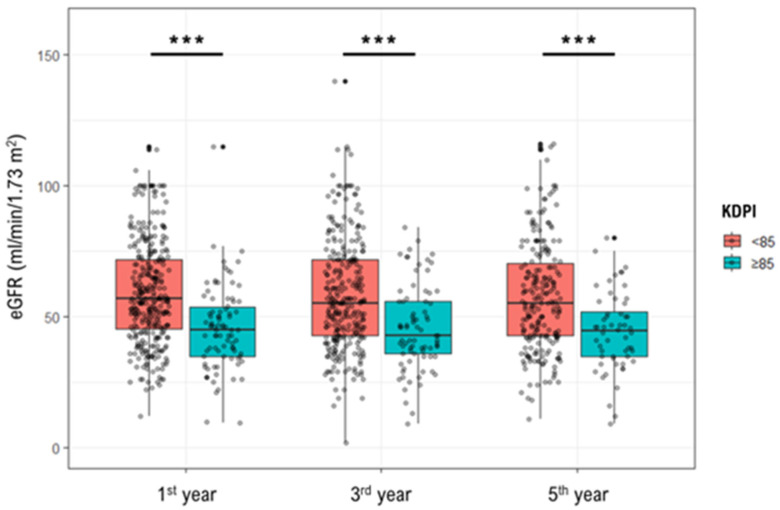
Boxplots depicting the effects of high KDPI on renal function at the 1st, 3rd, and 5th post-transplant year. KDPI ≥ 85 was associated with a significantly lower estimated glomerular filtration rate. *** *p*-value < 0.001.

Values of donor KDPI over 85 were associated with significantly lower eGFR in the first (45 vs. 57 mL/min/1.73 m^2^, *p*-value < 0.001), third (43 vs. 55 mL/min/1.73 m^2^, *p*-value < 0.001), and fifth (44.5 vs. 55 mL/min/1.73 m^2^, *p*-value < 0.001) post-transplant year (Figure 2). Table 2 presents the outcomes of the univariable and multivariable linear regression analysis. Specifically, after adjusting for recipient characteristics and immunosuppression, KDPI was associated with significantly lower eGFR in the first (β = −0.32, 95% CI: −0.38 to −0.26), third (β = −0.31, 95% CI: −0.38 to −0.25), and fifth year (β = −0.33, 95% CI: −0.41 to −0.26) after transplantation. Similarly, eGFR was inversely associated with KDRI and donor age in all multivariable models. Subgroup analysis indicated that the aforementioned associations remained significant only in SCD recipients.

### 3.4. Graft Survival

**Figure 3:**
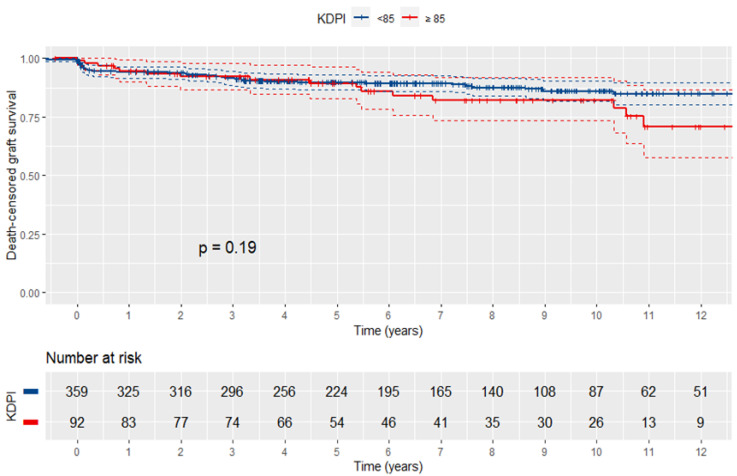
Kaplan–Meier curves of death-censored graft survival in patients with high and low KDPI. No significant difference was observed using the KDPI cut-off of 85.

Death-censored graft survival did not differ significantly among SCD and ECD recipients (log-rank *p*-value: 0.092). The outcomes of the Cox proportional hazards regression analysis regarding the association of KDPI, KDRI, and donor age with death-censored graft survival are presented in Table 3. After adjusting for covariates, KDPI was significantly linked to a 1% higher risk of graft failure per unit increase (HR: 1.01, 95% CI: 1.00 to 1.02, *p*-value: 0.016) in the overall population. Thus, patients with donor KDPI > 79 (4th quartile) were estimated to be at a threefold higher risk compared to those with donor KRPI < 25 (1st quartile) (HR: 3.07, 95% CI: 1.20 to 7.89). No significant difference was observed when patients with donor KDPI > 79 were compared to those with a donor KDPI of 25–54 (2nd quartile, *p*-value: 0.340) or 54–79 (3rd quartile, *p*-value: 0.969). A KDPI value above 85 (vs. <85) was not predictive of graft loss (log-rank *p*-value: 0.19; Figure 3). Correspondingly, graft survival was significantly worse among recipients with greater donor KDRI (HR: 2.21, 95% CI: 1.16 to 4.22) and age (HR: 1.03, 95% CI: 1.00 to 1.05). The unadjusted C-statistic was 0.54 for KDPI, 0.53 for KDRI, and 0.52 for donor age. The covariate-adjusted C-statistic was the same (0.69) in multivariable KDPI, KDRI, and donor age models. Subgroup analysis demonstrated no significant association of donor KDPI, KDRI, and age with death-censored graft failure risk when recipients of SCD and ECD were separately examined.

**Table 2:** Linear regression analysis outcomes evaluating the effects of KDPI, KDRI, and donor’s age on renal function at the first, third, and fifth post-transplant year.

	Crude Model	Model 1	Model 2	Model 3
	eGFR—1st year			
Overall				
KDPI ^†^	−0.33 (−0.38; −0.27)	−0.33 (−0.39; −0.28)	−0.33 (−0.39; −0.28)	−0.32 (−0.38; −0.26)
KDRI ^††^	−0.23 (−0.27; −0.19)	−0.24 (−0.28; −0.19)	−0.23 (−0.28; −0.19)	−0.22 (−0.27; −0.18)
Donor’s age ^†^	−0.65 (−0.75; −0.55)	−0.63 (−0.73; −0.53)	−0.64 (−0.75; −0.54)	−0.62 (−0.72; −0.52)
Standard-criteria donor				
KDPI ^†^	−0.38 (−0.48; −0.28)	−0.38 (−0.48; −0.28)	−0.38 (−0.48; −0.28)	−0.35 (−0.45; −0.24)
KDRI ^††^	−0.39 (−0.50; −0.29)	−0.39 (−0.50; −0.29)	−0.39 (−0.49; −0.28)	−0.35 (−0.46; −0.25)
Donor’s age ^†^	−0.66 (−0.82; −0.50)	−0.66 (−0.81; −0.50)	−0.66 (−0.82; −0.50)	−0.62 (−0.78; −0.45)
Expanded-criteria donor				
KDPI ^†^	−0.09 (−0.29; 0.11)	−0.10 (−0.31; 0.11)	−0.12 (−0.34; 0.09)	−0.11 (−0.33; 0.11)
KDRI ^††^	−0.05 (−0.12; 0.03)	−0.05 (−0.13; 0.03)	−0.06 (−0.15; 0.02)	−0.06 (−0.15; 0.03)
Donor’s age ^†^	−0.22 (−0.63; 0.18)	−0.30 (−0.73; 0.13)	−0.34 (−0.79; 0.10)	−0.33 (−0.80; 0.14)
	eGFR—3rd year			
Overall				
KDPI ^†^	−0.31 (−0.37; −0.25)	−0.31 (−0.38; −0.25)	−0.31 (−0.38; −0.25)	−0.31 (−0.38; −0.25)
KDRI ^††^	−0.21 (−0.26; −0.16)	−0.22 (−0.27; −0.17)	−0.22 (−0.27; −0.17)	−0.21 (−0.26; −0.16)
Donor’s age ^†^	−0.64 (−0.75; −0.52)	−0.65 (−0.76; −0.53)	−0.64 (−0.76; −0.53)	−0.63 (−0.75; −0.51)
Standard-criteria donor				
KDPI ^†^	−0.41 (−0.52; −0.29)	−0.40 (−0.52; −0.29)	−0.40 (−0.51; −0.28)	−0.40 (−0.52; −0.28)
KDRI ^††^	−0.43 (−0.55; −0.31)	−0.43 (−0.55; −0.31)	−0.42 (−0.54; −0.30)	−0.41 (−0.53; −0.29)
Donor’s age ^†^	−0.74 (−0.92; −0.55)	−0.73 (−0.91; −0.54)	−0.72 (−0.91; −0.54)	−0.71 (−0.89; −0.52)
Expanded-criteria donor				
KDPI ^†^	−0.10 (−0.34; 0.14)	−0.10 (−0.34; 0.15)	−0.16 (−0.40; 0.09)	−0.09 (−0.34; 0.16)
KDRI ^††^	−0.03 (−0.13; 0.06)	−0.04 (−0.13; 0.06)	−0.06 (−0.16; 0.04)	−0.03 (−0.13; 0.07)
Donor’s age ^†^	−0.43 (−0.89; 0.04)	−0.49 (−0.99; 0.04)	−0.57 (−1.08; −0.07)	−0.43 (−0.95; 0.09)
	eGFR—5th year			
Overall				
KDPI ^†^	−0.33 (−0.40; −0.27)	−0.34 (−0.41; −0.27)	−0.34 (−0.41; −0.27)	−0.33 (−0.41; −0.26)
KDRI ^††^	−0.23 (−0.28; −0.17)	−0.23 (−0.29; −0.18)	−0.23 (−0.29; −0.18)	−0.22 (−0.28; −0.17)
Donor’s age ^†^	−0.71 (−0.84; −0.59)	−0.72 (−0.85; −0.60)	−0.72 (−0.84; −0.59)	−0.70 (−0.84; −0.57)
Standard-criteria donor				
KDPI ^†^	−0.49 (−0.62; −0.36)	−0.49 (−0.62; −0.36)	−0.48 (−0.61; −0.34)	−0.47 (−0.60; −0.33)
KDRI ^††^	−0.49 (−0.63; −0.36)	−0.49 (−0.63; −0.36)	−0.48 (−0.62; −0.34)	−0.47 (−0.61; −0.33)
Donor’s age ^†^	−0.92 (−1.13; −0.72)	−0.92 (−1.13; −0.72)	−0.91 (−1.12; −0.71)	−0.88 (−1.09; −0.67)
Expanded-criteria donor				
KDPI ^†^	−0.19 (−0.46; 0.07)	−0.19 (−0.46; 0.09)	−0.24 (−0.51; 0.03)	−0.20 (−0.48; 0.08)
KDRI ^††^	−0.07 (−0.16; 0.03)	−0.06 (−0.17; 0.03)	−0.09 (−0.19; 0.01)	−0.07 (−0.17; 0.04)
Donor’s age ^†^	−0.44 (−0.93; 0.04)	−0.47 (−0.97; 0.04)	−0.46 (−0.96; 0.05)	−0.35 (−0.89; 0.18)

^†^ per unit increment; ^††^ per 0.01-unit increment. Model 1 adjusts for recipients’ age, sex, and body mass index. Model 2 adjusts additionally for recipients’ dialysis vintage and EPTS score. Model 3 adjusts additionally for induction and maintenance immunosuppression. Data presented as *β* (95% confidence intervals). Bold text indicates statistical significance. KDPI: Kidney Donor Profile Index; KDRI: Kidney Donor Risk Index; eGFR: estimated glomerular filtration rate.

### 3.5. Overall Survival

The results of the Cox proportional hazards regression analysis of overall patient survival are provided in Table A2. KDPI was not predictive of patient survival, neither as a continuous variable (HR: 1.00, 95% CI: 0.99–1.01) nor after applying the threshold value of 85 (HR: 0.83, 95% CI: 0.46–1.49). Similarly, overall survival was not significantly associated with donor KDRI (HR: 1.13, 95% CI: 0.63–2.02). Subgroup analysis did not indicate a significant link between KDPI/KDRI and patient survival in SCD and ECD transplantations.

### 3.6. Recipient Pairs Transplanted from the Same Donor

**Figure A1:**
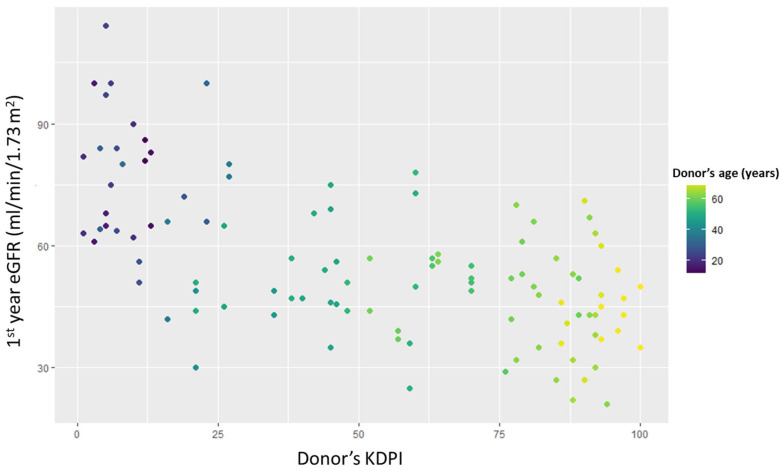
Scatterplot of 1st year-estimated glomerular filtration rate and donor’s KDPI among recipient pairs transplanted from the same donor.

Overall, the study included 62 pairs (124 recipients) who received a graft from the same deceased donor. Of them, during the follow-up, 16 recipients experienced graft loss; 54 pairs remained with functioning grafts, while graft loss was observed in both recipients in only one pair. Kidney function in the first year was inversely associated with donor’s KDPI (β: −0.35, *p*-value < 0.001) (Figure A1). The median difference of eGFR in the 1st post-transplant years between the recipients from the same pair was 9.50 mL/min/1.73 m^2^ (IQR: 2 to 19). This difference was not significantly associated with donor’s KDPI (*p*-value: 0.352).

## 4. Discussion

The present study retrospectively examined the prognostic value of KDRI and KDPI in a Greek transplant cohort, evaluating both SCD and ECD. The outcomes proposed that increasing KDPI is associated with significantly worse death-censored graft failure, with values in the fourth quartile of its distribution being linked to a threefold higher risk of graft loss compared to the first quartile. However, the prognostic value of KDPI was lost when donors were sub-grouped as SCD and ECD ones, while the overall discriminative ability was estimated to be modest. Donor age also emerged as an independent negative prognostic factor of graft survival, presenting a similar C-statistic to KDPI.

Regarding KDRI, it was observed that higher donor KDRI values were significantly associated with worse allograft survival. The median KDRI was calculated at 1.02, which is comparable to the median KDRI of the original US derivation cohort [6] (1.05) but was lower than the median values observed in other European studies [19,20,21,22]. Notably, the KDRI range of the present population was narrower (0.37 to 2.41) compared to the US cohort (0.5 to 4.2). The donors of the US cohort were younger than the donors of this Greek cohort (40 vs. 53 years) but included a significantly higher percentage of African Americans, hepatitis C-positive donors, and donors after circulatory death. As a result, it is anticipated that in the present population, a significant amount of the KDRI variability reflects differences in the donor’s age, explaining partially the similar discriminative ability of donor’s KDRI and age concerning graft loss.

The KDPI cut-off of 85 has been arbitrarily chosen since 15% of US deceased donors were expected to meet the ECD definition [6]. In the present cohort, ECD constituted 36.3% of the donor population, while nearly half of them (46.9%) had KDPI values below 85. The analysis demonstrated that the KDPI threshold of 85 was not predictive of graft failure, neither in the overall cohort nor among ECD transplantations. This finding is in line with the limited discriminative ability of the marker, suggesting that a substantial difference in KDPI between two grafts is needed to enable the prediction of worse long-term survival.

Similar outcomes regarding the prognostic value of KDPI were derived from other studies on the European population. Specifically, the analysis of a German transplant cohort [19] suggested that although higher KDPI values were linked to worse graft survival, its ability to predict graft failure remained limited for individuals (C-statistic: 0.62). In addition, a study using Eurotransplant Network Information System (ENIS) data showed that KDPI over 85 was associated with worse graft survival compared to KDPI below 35, although no single threshold could be defined to effectively discriminate the quality of grafts [20]. The analysis of a Spanish cohort proposed a 3% higher graft failure risk per unit increase of KDPI, resulting in a moderate predictive ability of KDPI, similar to that of donor age alone [21].

We observed that DGF was associated with significantly higher KDPI values among SCD, but not among ECD transplantations. This was probably related to the wider range of KDPI values in this subgroup, most likely related to the donor age. A similar finding was obtained by the study of Jun et al. [23], suggesting a significant link between KDPI and DGF risk only with donors ages below 60 years. Furthermore, KDPI was found to be predictive of significantly lower eGFR in the first, third, and fifth post-transplant years, although the index lost its prognostic value when ECD were separately examined. Our findings align with other studies having shown a significant association between KDPI and eGFR in the first [20] and in the first and fifth post-transplant year [22]. The present analysis indicated no association of donors’ KDRI, KDPI, or age with overall patient survival, which contrasts the findings of certain prior studies. This discrepancy may be based on donor differences (i.e., the inclusion of donors after circulatory death), as well as on the different recipient characteristics that are linked to high KDPI values in each cohort.

One of the main factors that contributed to the transition from the ECD/SCD dichotomy to the new allocation system, including KDPI/KDRI in the USA, was the ‘longevity matching’ concept in view of discard-rate decrease [24]. In a European prospective single-center study, Philipse et al. [25] reported that implementing the KDRI in their decision-making process increased the transplantation rate by 26%. However, the median KDRI of the transplant kidneys was 0.97 and only one patient received a deceased donor kidney with KDPI > 85%.

The development of KDPI is considered an important improvement over the dichotomized classification of donors to SCD ones, as it represents a more detailed and descriptive tool, able to reflect a greater amount of variability of donor characteristics. However, data from various European cohorts converge toward the conclusion that KDPI has limited efficacy in predicting long-term graft survival [19,20,21,22]. The observed discrepancy may be explained by the differences in donor and recipient characteristics between European populations and the original US cohort, as well as by time trends in transplantation practices since KDPI was derived. In this context, an analysis of ERA-EDTA registry between 2005 and 2015 demonstrated a 1.3% annual increase in KDRI, reflecting the inclusion of donors with greater age and higher percentages of hypertension, as well as the rising rates of donation after circulatory death. Nonetheless, 5-year transplantation outcomes remained unchanged, probably due to improvements in transplantation care and optimization of immunosuppression protocols [26].

The main strength of the present study relies on its long follow-up period, enabling the assessment of hard outcomes, such as graft failure and patient survival. A variety of multivariable models were applied, aiming to limit the effects of potential donor and recipient-specific confounders. Specifically, possible confounders were sequentially added to models, aiming to test the robustness of the estimated effects when different recipient factors were taken into account. However, the interpretation of the findings is limited by the retrospective observational nature of the study since residual confounding cannot be safely excluded. It should be also noted that it was a single-center study with a moderate sample size, which may jeopardize the generalizability of the results. Lastly, the lack of ethnicity variety and the exclusion of donors after circulatory death may have influenced the observed outcomes by limiting the variability of KDPI due to the aforementioned factors.

## 5. Conclusions

Τhe present analysis of a Greek transplant cohort provides evidence that although KDRI and KDPI are predictive of kidney function, they offer limited efficacy in accurately discriminating grafts with worse long-term survival. Therefore, the KDRI and KDPI scores may serve as adjunct clinical tools for the prediction of transplantation outcomes providing prognostic information about graft function, although they may be insufficient to guide decisions regarding kidney graft allocation in Greece. Further studies are needed to verify these findings, while large-scale cohorts may focus on the development of a modified prognostic index tailored to the Greek population. The derivation of an adapted KDPI may enhance its ability to predict transplantation outcomes, although whether such a marker would be able to ameliorate decision-making and optimize organ utilization remains to be investigated.

## Figures and Tables

**Table 1:** Baseline patients’ characteristics.

Variable	Overall	Expanded-Criteria Donor		*p*-Value
		Yes	No	
Donors				
Patients no.	394	143	251	
Age (years)	53 [39.3–61]	64 [61–69]	45 [30.5–52.5]	**<0.001**
Male sex	225 (57.1%)	69 (48.3%)	156 (62.2%)	**0.010**
BMI (kg/m^2^)	26.2 [24.2–28.6]	26.6 [24.8–29.5]	26.2 [23.7–27.8]	**0.006**
Diabetes mellitus	26 (6.6%)	20 (14.0%)	6 (2.4%)	**<0.001**
Hypertension	117 (29.7%)	79 (55.2%)	38 (15.1%)	**<0.001**
Serum creatinine (mg/dL)	0.80 [0.60–1.00]	0.83 [0.63–1.10]	0.80 [0.60–1.07]	0.270
KDPI	54 [26–79]	86 [76–92]	33 [16–52]	**<0.001**
KDRI	1.02 [0.78–1.34]	1.48 [1.29–1.64]	0.84 [0.70–1.01]	**<0.001**
Recipients				
Patients no.	456	169	287	
Age (years)	52 [43–59]	54 [45–61.8]	51 [42–59]	**<0.001**
Male sex	264 (57.9%)	95 (56.2%)	169 (58.9%)	0.646
BMI (kg/m^2^)	25.0 [23.0–27.7]	25.0 [22.8–27.2]	24.8 [23.0–27.7]	0.738
Diabetes mellitus	25 (5.5%)	9 (5.3%)	16 (5.6%)	1
Dialysis vintage (years)	7 [5–9]	7 [5–8]	7 [5–9]	0.768
EPTS score	37 [21–57]	41 [22–64]	34.5 [18.8–57]	0.072
Induction therapy				
*Basiliximab*	346 (75.9%)	107 (63.3%)	239 (83.3%)	**<0.001**
*Basiliximab + Rituximab*	23 (5.0%)	13 (7.7%)	10 (3.5%)	
*Daclizumab*	37 (8.1%)	19 (11.2%)	18 (6.3%)	
*Antithymocyte globulin*	50 (11.0%)	30 (17.8%)	20 (7.0%)	
Maintenance immunosuppression				
*MMF/CNI/Corticosteroids*	398 (87.3%)	136 (80.5%)	262 (91.3%)	**<0.001**
*MMF/mTORi/Corticosteroids*	1 (0.2%)	0 (0.0%)	1 (0.3%)	
*mTORi/CNI/Corticosteroids*	23 (5.0%)	9 (5.3%)	14 (4.9%)	
*MMF/Corticosteroids*	32 (7.0%)	24 (14.2%)	8 (2.8%)	
Delayed graft function	169 (37.1%)	78 (46.2%)	91 (31.7%)	0.558
eGFR—1st year (mL/min/1.73 m^2^)	54 [42.3–68]	46.7 [37–56.8]	59.5 [47–75]	**<0.001**
eGFR—3rd year (mL/min/1.73 m^2^)	52 [41–70]	46 [37–56]	57 [43–72]	**<0.001**
eGFR—5th year (mL/min/1.73 m^2^)	52 [41–67]	46 [35–56]	59 [43–76]	**<0.001**
Graft loss	58 (12.7%)	28 (16.6%)	30 (11.9%)	0.086
Death	89 (19.5%)	34 (20.1%)	55 (19.2%)	0.942
Follow-up period (years)	6.3 [3.6–10]	7.5 [4.5–10.6]	7.3 [4.03–10.2]	0.965

Data presented as median [interquartile range] or number (column percentage); BMI: body mass index; KDPI: Kidney Donor Profile Index; KDRI: Kidney Donor Risk Index; EPTS: estimated post-transplant survival; MMF: mycophenolate mofetil; mTORi: mammalian target of rapamycin inhibitor; CNI: calcineurin inhibitor; eGFR: estimated glomerular filtration rate. Bold text indicates statistical significance.

**Table 3:** Cox proportional hazards regression analysis evaluating the effects of KDPI, KDRI, and donor’s age on death-censored graft survival.

	Death-Censored Graft Loss			
	Crude Model	Model 1	Model 2	Model 3
Overall				
KDPI ^†^	1.01 (0.998–1.02)	1.01 (1.00–1.02)	1.01 (1.00–1.02)	1.01 (1.00–1.02)
KDPI (Q4 vs. Q1)	1.87 (0.87–4.03)	2.68 (1.09–6.59)	2.91 (1.14–7.42)	3.07 (1.20–7.89)
KDPI ≥ 85	1.47 (0.82–2.61)	1.79 (0.99–3.21)	1.58 (0.84–2.96)	1.53 (0.81–2.88)
KDRI ^†^	1.65 (0.90–3.02)	2.37 (1.25–4.47)	2.23 (1.16–4.31)	2.21 (1.16–4.22)
KDRI (Q4 vs. Q1)	1.80 (0.86–3.79)	2.84 (1.24–6.51)	3.08 (1.21–7.87)	3.32 (1.29–8.53)
Donor’s age ^†^	1.01 (0.99–1.02)	1.02 (0.999–1.04)	1.02 (1.00–1.04)	1.03 (1.00–1.05)
Standard-criteria donor				
KDPI ^†^	1.00 (0.99–1.02)	1.01 (0.99–1.03)	1.01 (0.99–1.03)	1.01 (0.99–1.03)
KDPI (Q4 vs. Q1)	1.51 (0.54–4.24)	1.88 (0.66–6.10)	1.99 (0.59–6.76)	2.08 (0.60–7.19)
KDPI ≥ 85	-	-	-	-
KDRI ^†^	0.98 (0.18–5.29)	1.97 (0.33–11.82)	2.82 (0.43–18.61)	2.90 (0.41–20.46)
KDRI (Q4 vs. Q1)	1.31 (0.45–3.77)	2.20 (0.65–7.40)	2.21 (0.65–7.50)	2.34 (0.68–8.04)
Donor’s age ^†^	1.00 (0.97–1.02)	1.00 (0.97–1.03)	1.01 (0.98–1.05)	1.01 (0.98–1.05)
Expanded-criteria donor				
KDPI ^†^	1.00 (0.97–1.03)	1.00 (0.97–1.03)	1.00 (0.96–1.03)	0.99 (0.96–1.02)
KDPI (Q4 vs. Q1)	1.07 (0.41–2.76)	1.12 (0.43–2.91)	0.98 (0.36–2.65)	0.85 (0.31–2.30)
KDPI ≥ 85	1.11 (0.52–2.36)	1.14 (0.53–2.43)	1.01 (0.46–2.22)	0.81 (0.37–1.78)
KDRI ^†^	1.40 (0.43–4.53)	1.62 (0.47–5.55)	1.42 (0.39–5.20)	1.03 (0.29–3.71)
KDRI (Q4 vs. Q1)	1.33 (0.50–3.58)	1.44 (0.53–3.87)	1.26 (0.45–3.57)	1.13 (0.40–3.18)
Donor’s age ^†^	1.00 (0.94–1.06)	1.02 (0.95–1.09)	1.01 (0.95–1.08)	1.01 (0.94–1.08)

^†^ per unit increment; Q1: 1st quartile; Q4: 4th quartile. Model 1 adjusts for recipients’ age, sex, and body mass index. Model 2 adjusts additionally for recipients’ dialysis vintage and EPTS score. Model 3 adjusts additionally for induction and maintenance immunosuppression. Data presented as hazard ratio (95% confidence intervals). Bold text indicates statistical significance. KDPI: Kidney Donor Profile Index; KDRI: Kidney Donor Risk Index.

## Data Availability

Data are available upon reasonable request.

## Appendix A

**Table A1:** Donor characteristics and coefficients used for KDRI calculation.

Donor Characteristics	Category	Component
Age (years)	18–50	0.0128 × (Age − 40)
	<18	−0.0194 × (Age − 18)
	>50	0.0107 × (Age − 50)
Height (cm)	All	−0.0464 × (Height − 170)/10
Weight (kg)	<80	−0.0199 × (Weight − 80)/5
Ethnicity	African American	0.1790
Hypertension	Yes	0.1260
Diabetes mellitus	Yes	0.1300
Death cause	Cerebrovascular accident	0.0881
Serum creatinine (mg/dL)	<1.5	0.2200 × (Creatinine − 1)
	>1.5	−0.2090 × (Creatinine − 1.5)
Hepatitis C status	Positive	0.2400
Donation after Circulatory Death	Yes	0.1330

**Table A2:** Cox proportional hazards regression analysis evaluating the effects of KDPI, KDRI, and donor’s age on overall patient survival.

	Overall Survival			
	Crude Model	Model 1	Model 2	Model 3
Overall				
KDPI ^†^	1.00 (0.99–1.01)	1.00 (0.99–1.01)	1.00 (0.99–1.01)	1.00 (0.99–1.01)
KDPI (Q4 vs. Q1)	1.42 (0.77–2.64)	1.33 (0.70–2.52)	1.60 (0.83–3.06)	1.25 (0.63–2.46)
KDPI ≥ 85	0.97 (0.56–1.68)	0.84 (0.48–1.47)	0.89 (0.50–1.57)	0.83 (0.46–1.49)
KDRI ^†^	1.30 (0.77–2.21)	1.08 (0.63–1.84)	1.26 (0.72–2.18)	1.13 (0.63–2.02)
KDRI (Q4 vs. Q1)	1.35 (0.74–2.45)	1.13 (0.61–2.11)	1.25 (0.66–2.37)	1.10 (0.57–2.13)
Donor’s age ^†^	1.01 (0.998–1.03)	1.00 (0.99–1.02)	1.01 (0.995–1.02)	1.01 (0.99–1.02)
Standard-criteria donor				
KDPI ^†^	1.00 (0.99–1.02)	1.00 (0.99–1.01)	1.00 (0.99–1.02)	1.00 (0.99–1.02)
KDPI (Q4 vs. Q1)	1.26 (0.60–2.65)	1.10 (0.50–2.45)	1.13 (0.50–2.53)	1.06 (0.45–2.48)
KDPI ≥85	-	-	-	-
KDRI ^†^	1.22 (0.35–4.23)	1.03 (0.27–3.85)	1.14 (0.29–4.43)	0.99 (0.22–4.42)
KDRI (Q4 vs. Q1)	1.16 (0.54–2.49)	1.02 (0.44–2.37)	1.12 (0.48–2.66)	1.01 (0.40–2.54)
Donor’s age ^†^	1.01 (0.99–1.03)	1.01 (0.99–1.03)	1.01 (0.99–1.03)	1.01 (0.99–1.03)
Expanded-criteria donor				
KDPI ^†^	0.99 (0.97–1.02)	0.98 (0.96–1.01)	0.99 (0.96–1.02)	0.99 (0.96–1.02)
KDPI (Q4 vs. Q1)	0.97 (0.37–2.56)	0.73 (0.26–1.98)	0.84 (0.30–2.34)	0.96 (0.32–2.87)
KDPI ≥85	0.61 (0.31–1.21)	0.52 (0.26–1.05)	0.55 (0.27–1.12)	0.55 (0.26–1.17)
KDRI ^†^	0.83 (0.28–2.43)	0.48 (0.17–1.37)	0.60 (0.20–1.77)	0.74 (0.24–2.32)
KDRI (Q4 vs. Q1)	1.33 (0.50–3.58)	1.44 (0.53–3.87)	1.26 (0.45–3.57)	1.13 (0.40–3.18)
Donor’s age ^†^	1.01 (0.96–1.07)	0.97 (0.92–1.03)	0.97 (0.92–1.02)	0.98 (0.92–1.05)

^†^ per unit increment; Q1: 1st quartile; Q4: 4th quartile. Model 1 adjusts for recipients’ age, sex and body mass index. Model 2 adjusts additionally for recipients’ dialysis vintage and EPTS score. Model 3 adjusts additionally for induction and maintenance immunosuppression. Data presented as hazard ratio (95% confidence intervals). KDPI: Kidney Donor Profile Index; KDRI: Kidney Donor Risk Index.
